# Thin-Film Deposition: From Fundamental Research to Applications

**DOI:** 10.3390/mi15121503

**Published:** 2024-12-17

**Authors:** Laura Patricia Rivera Reséndiz, José Guadalupe Quiñones Galván

**Affiliations:** Departamento de Física, CUCEI Universidad de Guadalajara, Blvd. Marcelino García Barragán, Guadalajara 44430, Mexico; laura.rivera@academicos.udg.mx

In recent decades, we have witnessed incredible advancements in technology. Since the invention of the transistor by John Bardeen, Walter Brattain and William Shockley in the 1940s [1], electronic devices have considerably reduced in size, allowing for better performance of miniaturized devices. This reduction has mainly been driven by the continuous development of thin-film deposition techniques or the improvement of existing methods. These factors together, with theoretical studies, have led to the accelerated growth of materials that can be applied in a huge variety of fields, from electronics to energy storage and conversion, biomedicine, ambient remediation, etc. [2].

This Special Issue focusses on fundamental research on thin films and their applications, including topics such as microheating, ferroelectricity, solar cells, polymers, batteries, thermoelectrics, chemical etching, extreme UV optics, semiconductors and RF applications. This Special Issue consist of 11 contributions, including a review paper.

Qu et al. (contribution 1) studied the influence of sputtering deposition conditions on tungsten (W) thin films’ properties. They found that the temperature coefficient of resistance, a key parameter for microheating applications, can be modulated by changing the deposition pressure and temperature. Moreover, they used an optimized W thin film as a microheater in a RF phase-change switch, obtaining higher voltage values when compared with similar devices.

Gao et al. (contribution 2) analyzed the effects of oxygen pressure on the mechanical properties of samarium (Sm) doped BiFeO_3_ thin films grown via pulsed laser deposition. Their results showed a correlation between the microstructure and nanomechanical properties of the films, demonstrating that grain boundaries can inhibit the dislocation movements in Sm-doped bismuth ferrite.

Using DC magnetron sputtering deposition, Rosales Medina et al. (contribution 3) achieved nanocolumnar growth of TiO_2_ bilayers for use as a high-quality electron transport layer. This columnar growth was achieved by developing a glancing angle deposition configuration. The authors studied the influence of sputtering power on the crystalline quality of the films. These layers are highly interesting for perovskite solar cell applications.

Cutroneo el al. (contribution 4) grew titanium thin films on cyclic olefin copolymer (COC) foils through pulsed laser deposition using masks for selective deposition. They explored changes in the wettability of the polymer following its modification with a Ti layer, finding that the native hydrophilicity decreased for the Ti-covered polymers. This reduction could allow for the elimination of restrictions on the use of COC in a number of applications.

Lobinsky et al. (contribution 5) developed Mn_3_[Fe(CN)_6_]_2_·nH_2_O nanosheets for use as a novel analog of Prussian blue in high-performance ion batteries. These nanosheets have potential to overcome the poor electrical conductivity and stability of the Prussian blue analogs proposed so far. By using successive ionic layer deposition, Lobinsky et al. reported exceptional cathode performance of the synthesized nanosheets, with excellent specific capacitance values.

Ahn et al. (contribution 6) used trapezoid-patterned sapphire substrates and a gallium nitride (GaN) layer to fabricate air tunnel structures through the in situ carbonization of a photoresist layer, enabling rapid chemical lift-off. The GaN layer was epitaxially grown via a metal–organic chemical deposition technique. Their results showed successful separation of the substrate from the GaN layer, overcoming the limitations associated with the laser lift-off technique.

Reyes-Verdugo et al. (contribution 7) used the pulsed laser deposition technique to grow Bi_2_Te_3_ thin films. They implemented a novel methodology consisting of the simultaneous ablation of independent Bi and Te targets that allows the combination of reactive Bi and Te plasmas. One of their main findings was that stoichiometric Bi_2_Te_3_ thin films can be obtained more easily than when using stoichiometric compound pellets as targets for the ablation process.

Liu et al. (contribution 8) performed a comparative study of a Mo/Si multilayer microstructure. They deposited layers using the sputtering technique on curved substrates used for extreme ultraviolet astronomy applications. The microstructural properties of the multilayer systems deposited using a shadow mask were compared with those of layers grown without the mask. It was found that the mask did not have a considerable effect on the morphological and microstructural properties of the films.

Another contribution of Cutroneo et al. (contribution 9) demonstrates that irradiating with 10 MeV carbon ions, which is an energy value significantly lower than the hundreds of MeV typically used, can induce customized pore locations without overlapping in polyethylene terephthalate (PET) foils.

Chen et al. (contribution 10) studied the effect of substrate temperature on the radio frequency sputtering deposition of nickel oxide (NiO) films. Their main findings included crystalline improvement with increasing temperature. This improvement affected the mechanical properties of the films by increasing their hardness and elastic modulus.

Finally, Qu et al. (contribution 11) presented a review on GeTe-based radio frequency phase-change switches. They performed a detailed exploration of thin-film systems based on GeTe thin films. Additionally, they conducted comparative analyses of direct and indirect heating structures, which allowed them to select the materials for each layer of the different devices, along with their dimensions, based on the thicknesses of the layers within the explored switching devices.
